# Feedstock-Dependent Water Sorption and Retention of Citric-Acid-Modified Lignocellulosic Biogels Under Simulated Climatic Conditions

**DOI:** 10.3390/gels12090820

**Published:** 2026-09-07

**Authors:** Tomáš Holeček, Michaela Filipi, Ivana Tomášková, Karolina Resnerová, Jan Macků, Kateřina Hájková

**Affiliations:** 1 Faculty of Forestry and Wood Sciences, Czech University of Life Sciences Prague, Kamýcká 129, 165 00 Prague, Czech Republic; holecekt@fld.czu.cz (T.H.); tomaskova@fld.czu.cz (I.T.); resnerovak@fld.czu.cz (K.R.); macku@fld.czu.cz (J.M.); 2 Faculty of Chemical Technology, University of Pardubice, Studentská 95, 532 10 Pardubice, Czech Republic; michaela.filipi@upce.cz

**Keywords:** biomass composition, agricultural residues, soil conditioner, water-holding capacity, FTIR spectroscopy, citric-acid modification

## Abstract

Increasing drought frequency and irregular precipitation patterns highlight the need for sustainable soil water management. In this study, lignocellulosic biogels were prepared from Norway spruce and silver birch sawdust and from rapeseed and poppy straw using a 10 wt.% citric acid solution, with a fixed addition of 5 mL of 80% lactic acid as a processing modifier. Two formulations differing in the volume of citric acid solution (180 or 250 mL per 12 g of dry feedstock), and consequently in both the liquid-to-solid ratio and absolute citric acid dose, were evaluated. Biomass chemical composition and density were determined, the amount of water absorbed by soil–biogel mixtures containing approximately 3.1% dry biogel relative to dry soil mass was measured gravimetrically after saturation and free drainage and water-loss kinetics of the separately saturated biogels were monitored under cyclic climatic conditions ranging from 12 to 35 °C and 25 to 75% relative humidity. FTIR spectroscopy was used to compare cellulose isolated from each feedstock with the corresponding biogels prepared using both the 180 and 250 mL formulations. Biogel performance depended on feedstock composition and physical characteristics. Among the tested formulations, the poppy-derived 180 mL biogel produced the highest water absorption in the soil–biogel system, whereas spruce-derived biogels exhibited more stable residual water content during prolonged desorption. The poppy-derived 180 mL formulation increased water absorption in the soil–biogel system from 88.46 g in untreated soil to 122.86 g, corresponding to an increase of 38.9%. FTIR analysis indicated an increased contribution of carbonyl-containing structures consistent with incorporation of the acid modifiers and possible ester formation; however, the spectra did not provide direct evidence of crosslinking density. Increasing the citric acid solution volume did not consistently improve water absorption by the soil–biogel system or water-loss behavior of the isolated biogels. These laboratory results demonstrate a feedstock-dependent interaction with formulation and provide a basis for further evaluation of lignocellulosic residues as renewable water-retaining soil amendments under soil–plant and field conditions.

## 1. Introduction

Increasing drought frequency, irregular precipitation, rising temperatures, and higher evapotranspiration are reducing soil water availability in agricultural and forest ecosystems. Water-retaining soil amendments are therefore being considered as a complementary measure to conventional practices such as mulching, irrigation management, and increasing soil organic matter. Hydrogels and other water-retaining polymeric materials can temporarily store water and release part of it as the surrounding substrate dries, thereby potentially increasing soil water retention during periods of limited rainfall [1,2].

The behavior of hydrogels in soil depends not only on their maximum swelling capacity but also on how rapidly the absorbed water is lost during drying. Polymer composition, network structure, soil properties, temperature, relative humidity, and ionic conditions all affect sorption and desorption. A material with high initial water uptake may therefore show only limited long-term retention if a large proportion of the absorbed water is weakly bound. Recent studies on cellulose-based hydrogels confirm that differences in formulation influence both water uptake and subsequent water release in soil systems [3]. However, intrinsic swelling of an isolated material, water absorption by a soil-amendment mixture after drainage, and residual water content during drying represent different operational parameters and should not be interpreted interchangeably.

Most commercial superabsorbents are produced from synthetic polymers because they provide high swelling capacity and controllable properties. Their persistence in soil, however, has increased interest in materials derived from renewable resources. Cellulose, hemicelluloses, lignin, starch, alginate, and chitosan have all been investigated as components of bio-based water-retaining materials. Forestry and agricultural residues are particularly attractive because they combine the use of underutilized renewable feedstocks with the possibility of biomass-waste valorization [4,5]. Recent studies also show that whole lignocellulosic biomass can be used directly in soil-conditioning gels without complete separation of its individual polymer fractions [6]. Nevertheless, the performance of whole-biomass materials cannot be inferred directly from studies employing isolated cellulose, lignin, or other purified polymers.

Whole lignocellulosic feedstocks are chemically and structurally heterogeneous. Cellulose and hemicelluloses provide hydroxyl groups that interact with water and can participate in chemical modification, whereas lignin is less hydrophilic and may contribute to rigidity and resistance to structural collapse during drying. The resulting water uptake and retention therefore depend on the combined effects of cellulose, hemicelluloses, lignin, extractives, inorganic components, particle structure, and other feedstock-specific physical characteristics rather than on cellulose content alone [7,8,9]. These differences are particularly relevant when woody biomass is compared with agricultural residues, which often differ in their proportions of structural carbohydrates, lignin, extractives, ash, and in their particle-packing properties. Norway spruce and silver birch represent chemically distinct softwood and hardwood feedstocks, whereas poppy and rapeseed straw represent underutilized agricultural residues with substantially different non-cellulosic fractions. Their direct comparison therefore provides a deliberately broad compositional contrast rather than a comparison among taxonomically similar materials.

Citric acid is widely used as a comparatively environmentally acceptable modifier and potential crosslinking agent for polysaccharide-rich materials. Under appropriate thermal conditions, its carboxyl groups can react with hydroxyl groups of cellulose and other polysaccharides and form ester linkages [10,11,12]. This reaction can improve the stability of the material in water, but a higher amount of citric acid does not necessarily result in better sorption performance. Insufficient modification may produce a poorly stabilized structure, whereas excessive esterification or acid incorporation can restrict chain mobility and reduce swelling [13,14]. The response may also differ among feedstocks because the availability of reactive hydroxyl groups and the accessibility of the lignocellulosic matrix are not identical. However, an increased FTIR signal in the carbonyl region alone cannot distinguish covalently formed ester bonds from residual non-esterified carboxylic acids and therefore does not provide direct evidence of crosslinking density. Residual organic acids may additionally influence material acidity and water sorption behavior.

Previous studies have demonstrated that cellulose-, lignin-, and biomass-based hydrogels can improve soil hydration and delay water loss [3,6,15]. Nevertheless, most studies focus on a single purified biopolymer, one biomass source, or a hybrid system containing both natural and synthetic polymers. Direct comparisons of woody and agricultural lignocellulosic feedstocks processed under identical conditions remain limited. Moreover, quantitative comparisons among published materials are complicated by differences in feedstock processing, polymer concentration, amendment dose, soil type, water chemistry, saturation procedure, and the definition used for water absorption or retention. In addition, swelling is often evaluated under constant laboratory conditions, whereas materials applied to soil are exposed to daily changes in temperature and relative humidity. These fluctuations may influence evaporation, matrix contraction, and the proportion of water retained over time. Information on the desorption behavior of whole-biomass lignocellulosic biogels under cyclic climatic conditions is therefore still insufficient [1,2,16].

The present study compared citric-acid-modified lignocellulosic biogels prepared from Norway spruce and silver birch sawdust and from rapeseed and poppy straw. Two formulations differing in the volume of a 10 wt.% citric acid solution were evaluated. For 12 g of dry feedstock, either 180 or 250 mL of the solution was used, meaning that the two operational formulations differed simultaneously in solution volume, liquid-to-solid ratio, and absolute citric acid dose. Lactic acid was added at the same fixed amount to all formulations. The comparison was therefore designed to determine whether increasing the overall availability of the citric acid solution produced a consistent response across chemically different feedstocks, rather than to isolate the effect of a single reaction variable.

The chemical composition and selected physical characteristics of the starting feedstocks were related to the amount of water absorbed by soil–biogel mixtures after saturation and free drainage and to the water-loss behavior of separately saturated biogels under cyclic temperature and relative-humidity conditions. FTIR spectroscopy was additionally used to compare cellulose isolated from each feedstock with the corresponding biogels prepared using both formulations and to identify spectral changes consistent with the incorporation of carbonyl-containing acid components and possible ester formation.

The novelty of this study lies in the side-by-side comparison of two woody and two agricultural whole-biomass feedstocks processed using the same preparation procedure, combined with evaluation of both soil–biogel water absorption and prolonged biogel desorption under repeated climatic cycles. It was hypothesized that feedstock composition and physical characteristics would influence both initial water absorption in the soil–biogel system and subsequent water-loss behavior and that increasing the volume of citric acid solution would not affect all feedstocks in the same way.

## 2. Results and Discussion

### 2.1. Selected Chemical Composition of the Feedstocks

The selected chemical constituents of the four lignocellulosic feedstocks differed considerably (Table 1). Spruce contained the highest proportions of cellulose (47.90%) and lignin (28.90%), whereas birch contained 38.70% cellulose and 19.21% lignin. Poppy and rapeseed residues contained higher proportions of acetone-soluble extractives and ash than both woody feedstocks, while rapeseed showed the highest measured hemicellulose content. These compositional contrasts supported the selection of the four feedstocks for comparing wood and agricultural whole-biomass materials.

The selected feedstocks provided compositionally distinct lignocellulosic matrices for biogel preparation. Spruce was characterized by comparatively high cellulose and lignin contents, whereas the agricultural residues contained larger acetone-soluble and inorganic fractions. Rapeseed also showed the highest calculated hemicellulose content. These differences may influence the availability of hydrophilic functional groups and the response of the whole-biomass materials to acid treatment.

Cellulose provides hydroxyl groups that can participate in water binding and possible acid-mediated ester formation, while lignin may reduce swelling because of its lower hydrophilicity but contribute to structural stability during drying [7,8,9]. Hemicelluloses may also support water uptake because of their hydrophilic character and lower structural order. However, the contribution of the individual components cannot be isolated from the present whole-feedstock experiment, and the observed water behavior should therefore be interpreted as the combined response of all measured and unmeasured biomass constituents.

Wood density and the bulk density of agricultural residues were originally determined using different procedures and represent physically different quantities. They were therefore not treated as directly comparable explanatory variables and were excluded from the combined regression analysis. Because the internal morphology and quantitative porosity of the resulting biogels were not measured, differences in water absorption cannot be attributed directly to pore accessibility, matrix compactness, or porosity.

### 2.2. FTIR Characterization of Isolated Cellulose and Acid-Modified Whole-Biomass Biogels

**Figure 1:**
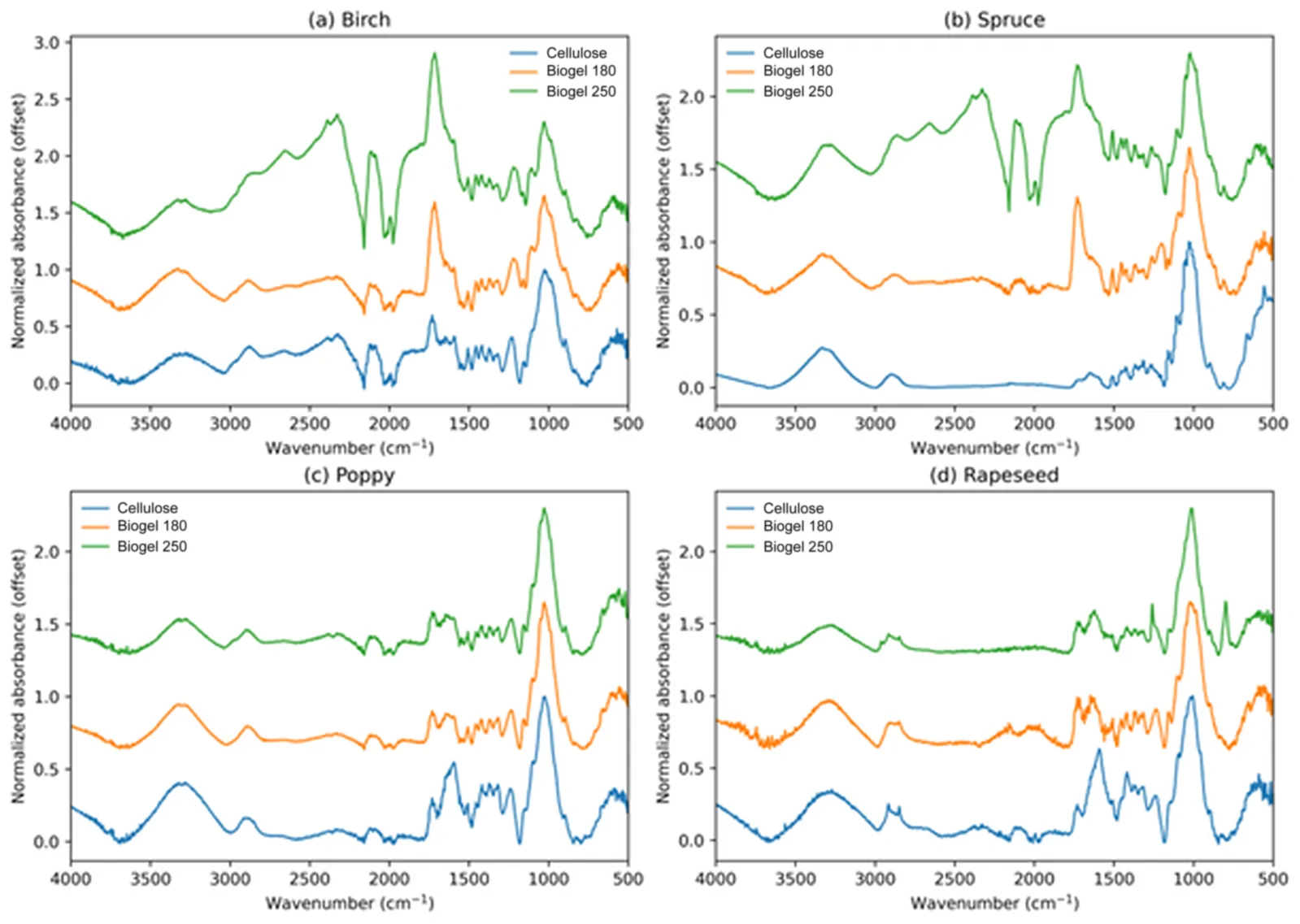
FTIR spectra of isolated cellulose and the corresponding acid-modified whole-biomass biogels prepared using 180 and 250 mL of 10 wt.% citric acid solution: (**a**) birch, (**b**) spruce, (**c**) poppy, and (**d**) rapeseed. Spectra were baseline-corrected, normalized, and vertically offset for clarity. The isolated cellulose spectra are shown as compositional references rather than as chemically equivalent untreated controls.

FTIR spectra of cellulose isolated from each feedstock were compared with those of the corresponding biogels prepared using 180 and 250 mL of 10 wt.% citric acid solution (Figure 1). All spectra were converted to absorbance, baseline-corrected and normalized to the polysaccharide C–O stretching region before graphical comparison. The principal bands considered in the interpretation are summarized in Table 2. Because isolated cellulose and the whole-biomass biogels differ fundamentally in chemical composition, this comparison was used only to describe spectral differences and not to attribute those differences exclusively to acid-induced reactions. Untreated whole-feedstock controls subjected to the same thermal treatment and washed biogel samples were not available.

**Figure 2:**
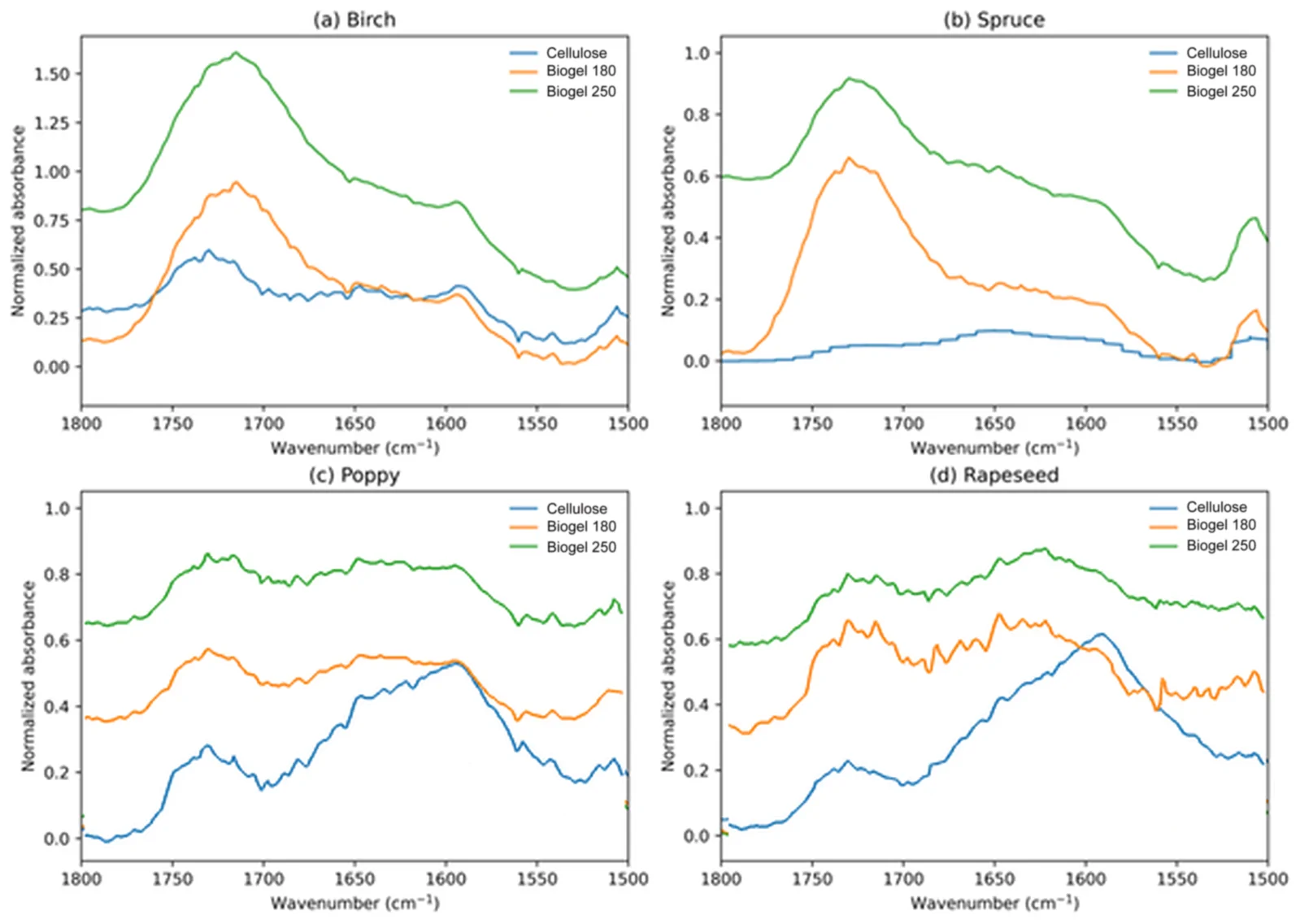
Detail of the FTIR carbonyl region (1800–1500 cm^−1^) for isolated cellulose and the corresponding 180 and 250 mL acid-modified whole-biomass formulations. Differences between isolated cellulose and the biogels include both feedstock-composition effects and possible treatment-induced changes.

The cellulose spectra were dominated by the broad O–H stretching band at 3600–3000 cm^−1^, C–H stretching near 2900 cm^−1^, and strong C–O and C–O–C vibrations in the fingerprint region. In the spectra of the acid-modified whole-biomass materials, the most relevant differences occurred in the carbonyl region between approximately 1760 and 1680 cm^−1^ (Figure 2). The response was feedstock dependent. Birch and spruce biogels showed a marked increase in carbonyl-region absorbance relative to the corresponding isolated cellulose, whereas the change was smaller for rapeseed and was not clearly enhanced for poppy.

These spectral differences indicate a greater relative contribution of carbonyl-containing structures in some whole-biomass biogels. However, they may originate from several overlapping sources, including native hemicelluloses, lignin and extractives, residual non-esterified citric and lactic acids, and possible ester bonds formed during thermal treatment. Consequently, the observed bands are consistent with the incorporation of the acid modifiers and possible ester formation but do not demonstrate the formation or density of a covalently crosslinked network.

To compare the relative contribution of absorbance in the carbonyl region semiquantitatively, a carbonyl index was calculated as the ratio of the baseline-corrected integrated absorbance in 1760–1680 cm^−1^ to that at 1080–1000 cm^−1^ (Table 3). The index was higher for the birch and spruce biogels than for their respective isolated-cellulose references and showed a smaller difference for rapeseed. In contrast, the poppy biogels showed values close to, or slightly below, the corresponding cellulose reference. The effect of the formulation was not uniform: the 250 mL variant produced the highest index for birch and rapeseed, whereas spruce showed a higher value in the 180 mL formulation.

Because the reference and biogel samples differed in overall chemical composition and only one spectrum was recorded per sample, the index was treated solely as a descriptive indicator of the relative carbonyl contribution. It was not subjected to statistical analysis and cannot be interpreted as a measure of esterification or crosslinking density.

The increase in the carbonyl region observed for several biogels agrees with previous studies on citric-acid-modified cellulose, in which bands near 1710–1740 cm^−1^ were assigned to ester carbonyl groups together with contributions from residual carboxylic groups introduced during thermal treatment [10,12,17]. Similar spectral changes have also been reported for citric-acid-crosslinked microbial cellulose and other polysaccharide hydrogels [18,19].

However, FTIR alone cannot distinguish esterified citric acid from non-esterified carboxyl groups. Because the present biogels were not subjected to a post-reaction washing step, and because lactic acid was also added during preparation, both acids may have contributed to the carbonyl-region absorbance. In addition, native carbonyl-containing constituents present in the whole feedstocks were absent or substantially reduced in the isolated-cellulose references. The spectra and carbonyl index are therefore interpreted as evidence of carbonyl-containing acid incorporation and possible chemical modification rather than as direct evidence or a quantitative measure of crosslinking density.

The absence of a consistent increase from 180 to 250 mL shows that the spectral response did not vary solely with the amount of citric acid solution used. The observed differences may reflect the combined effects of feedstock composition, residual acid content, moisture, sample–ATR contact, and possible ester formation.

The FTIR results should therefore be interpreted together with the water-holding and desorption data, because a stronger carbonyl contribution did not necessarily correspond to improved water absorption or slower water loss. Confirmation of covalent network formation would require chemically equivalent untreated controls, post-reaction washing, replicate spectra, and complementary analyses such as gel-fraction determination or another direct assessment of network formation.

### 2.3. Water Absorption by Soil–Biogel Systems After Free Drainage

The addition of lignocellulosic biogels increased the mass of water retained after saturation and free drainage in all soil–biogel systems compared with the untreated reference (Table 4). The dry biogel-to-dry-soil mass ratio ranged from approximately 3.13 to 3.19%, corresponding to an application rate of approximately 3.1 (*w*/*w*). The reference soil retained 88.46 ± 0.38 g of water, whereas the values obtained for the biogel-containing systems ranged from 97.30 ± 1.11 to 122.86 ± 2.07 g.

The highest amount of retained water was recorded for the poppy biogel prepared using 180 mL of citric acid solution, corresponding to an increase of 38.9% relative to the untreated soil. High values were also obtained for spruce 250 and poppy 250, which increased the retained water mass by 32.7 and 28.5%, respectively. These values describe the response of the complete soil–biogel system and should not be interpreted as the intrinsic swelling capacity of the isolated biogels.

The effect of the operational formulation differed among feedstocks. Increasing the volume from 180 to 250 mL increased the retained water mass in the spruce and rapeseed systems, whereas the 180 mL formulation performed better for birch and poppy. The difference between formulations was most pronounced for spruce, for which the 250 mL treatment retained approximately 15.34 g more water than the corresponding 180 mL treatment. In contrast, birch 250 retained approximately 9.42 g less water than birch 180.

Because the two formulations differed simultaneously in solution volume, liquid-to-solid ratio, absolute citric acid dose, and drying conditions, these differences cannot be attributed solely to the amount of citric acid.

A two-way ANOVA conducted for the eight biogel-containing treatments showed a significant effect of feedstock on the mass of water retained after free drainage, F(3, 32) = 269.37, *p* < 0.001, partial η^2^ = 0.962. The main effect of formulation (180 vs. 250 mL) was not significant, F(1, 32) = 2.23, *p* = 0.145, partial η^2^ = 0.065. However, a significant feedstock × formulation interaction was observed, F(3, 32) = 178.16, *p* < 0.001, partial η^2^ = 0.944, indicating that the difference between the two operational formulations depended strongly on the lignocellulosic feedstock. The untreated reference soil was excluded from the factorial analysis because it did not represent either level of the formulation factor. Accordingly, the analysis supports a feedstock-dependent formulation response but does not identify which individual processing variable caused that response.

The highest retained water mass was observed for poppy 180, although poppy did not contain the highest proportion of cellulose. Spruce, which had the highest cellulose content, reached its highest value only in the 250 mL formulation. Water absorption by the soil–biogel system was therefore not controlled by cellulose content alone. Hemicelluloses, lignin, extractives, accessibility of hydrophilic groups, residual organic acids, particle characteristics, and the extent of acid incorporation or possible chemical modification may also have contributed to the observed differences.

However, because these factors were not manipulated independently and biogel morphology was not measured, their individual contributions cannot be resolved from the present data.

The contrasting response of the two formulations shows that increasing the volume of citric acid solution did not automatically improve the amount of water retained by the soil–biogel system.

The higher-volume formulation simultaneously increased the liquid-to-solid ratio and the absolute citric acid dose and may also have altered mixing, mass transfer, residual acid content, and drying behavior. The present experimental design does not allow these effects to be separated. The FTIR results likewise showed no uniform relationship between the descriptive carbonyl index and water absorption; therefore, the observed formulation effects should not be interpreted as evidence of differences in crosslinking density.

All biogel treatments increased the amount of water retained by the tested soil by 10.0–38.9% at an application rate of approximately 3.1% (*w*/*w*). Quantitative comparison with previous hydrogels must nevertheless be made cautiously because published studies frequently report the equilibrium swelling of isolated materials rather than water absorption by a soil–biogel mixture. For example, Jong et al. [3] reported isolated-hydrogel swelling ratios of approximately 808–3563% and residual water-retention values of 9.24–33.47% after 96 h, depending on the plasticizer. Alsubaie et al. [6] reported equilibrium swelling values of approximately 400–700% for purified date-palm-derived hydrogels and evaluated a 0.5% soil amendment rate. Fihurka & Budnyak [15] obtained swelling capacities of up to 235 g g^−1^ for semisynthetic kraft-lignin/polyacrylamide hydrogels and tested a 2.5% addition to soil. These values are not directly equivalent to the present soil-system measurement, which did not determine the equilibrium swelling ratio of the isolated biogels. Consequently, superiority over previously reported hydrogels cannot be claimed. The distinguishing contribution of the present study is the direct comparison of four whole-biomass feedstocks under the same preparation and free-drainage conditions.

Within the limits of the single-soil laboratory experiment, all tested biogel additions increased the mass of water retained after saturation and free drainage relative to the untreated soil. The results identify feedstock selection and formulation as relevant factors for further material optimization but do not demonstrate improved plant water availability, reduced drought stress, or field performance. These potential applications require testing across different soils, water chemistries, amendment rates, and soil–plant systems.

### 2.4. Relative Water Retention Under Simulated Climatic Conditions

**Figure 3:**
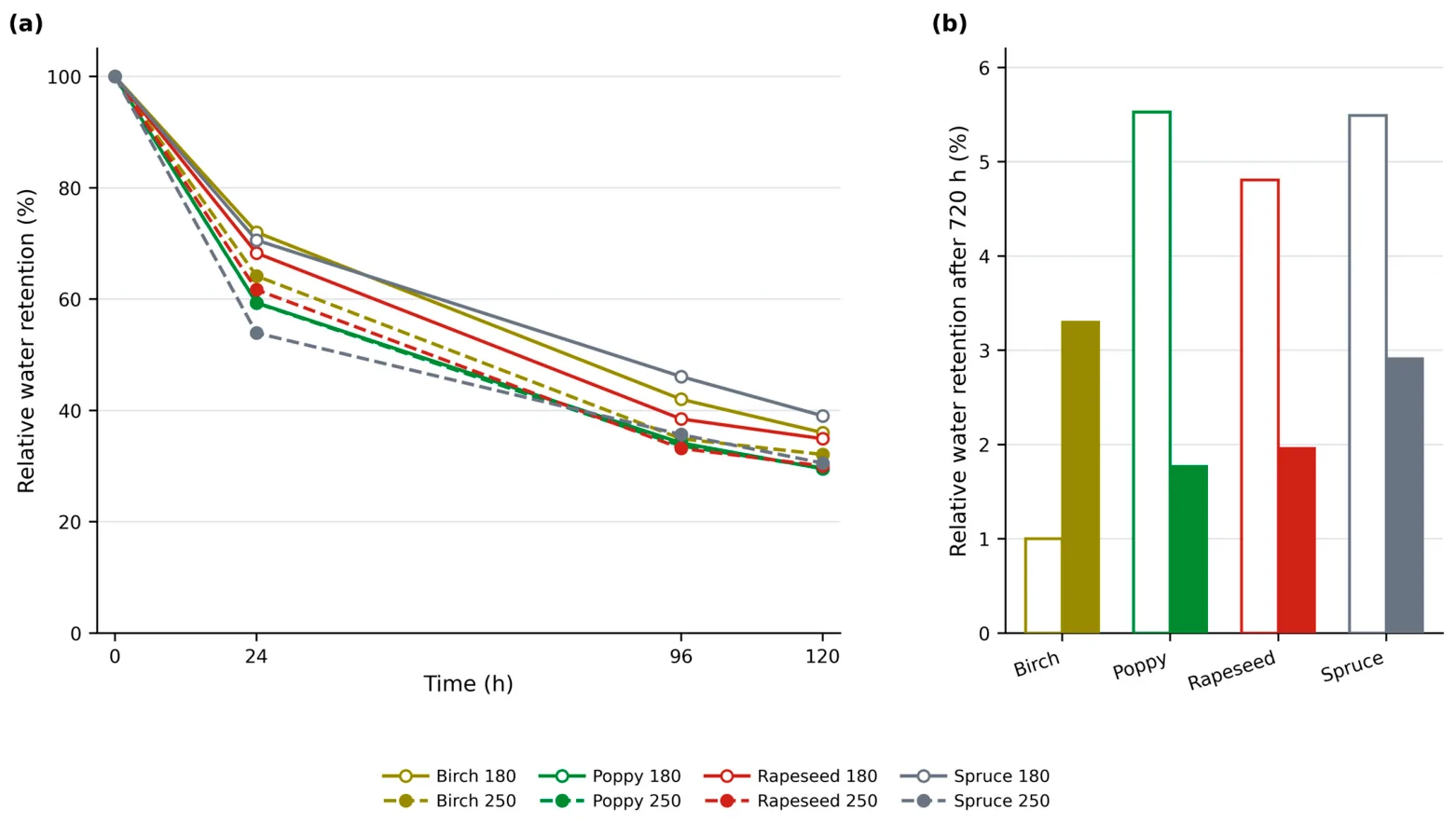
Relative water retention of citric-acid-modified lignocellulosic biogels under repeated climatic cycles: (**a**) relative retention during the initial 120 h and (**b**) residual retention after 720 h. Mean water-content values were normalized to the initial value of each formulation, which was set to 100%. Open symbols, solid lines, and open bars represent the 180 mL formulations; filled symbols, dashed lines, and filled bars represent the corresponding 250 mL formulations. Values represent normalized means of five replicate measurements (*n* = 5).

The relative water retention of separately saturated lignocellulosic biogels was evaluated under cyclic temperature and relative-humidity conditions. Mean water-content values were normalized to the initial value of each formulation, which was set to 100%. The retention profiles during the initial 120 h and the residual retention after 720 h, corresponding to 30 repeated 24 h climatic cycles, are presented in Figure 3. No intermediate weighing was performed between 120 and 720 h.

All formulations showed their largest decrease in relative water retention during the first 24 h. After this period, the remaining water ranged from approximately 53.9% for spruce 250 to 72.0% for birch 180. Spruce 180 and rapeseed 180 retained approximately 70.6 and 68.3%, respectively, whereas both poppy formulations retained approximately 59% of their initial water content. The first 24 h therefore represented the principal water-loss stage under the applied climatic regime.

Between 24 and 120 h, water loss continued more gradually. Spruce 180 showed the highest relative retention at both 96 h (approximately 46.1%) and 120 h (approximately 39.0%). Birch 180 retained approximately 42.0% after 96 h and 36.0% after 120 h, whereas rapeseed 180 retained approximately 38.5 and 34.9%, respectively. The remaining formulations showed relative retention values of approximately 33–36% after 96 h and 30–32% after 120 h.

After 720 h, only a small proportion of the initially absorbed water remained in all formulations. Poppy 180 and spruce 180 showed the highest residual retention, both approximately 5.5%, followed by rapeseed 180 at approximately 4.8%. Among the 250 mL formulations, residual retention ranged from approximately 1.8% for poppy 250 to 3.3% for birch 250. Birch 180 showed the lowest residual retention, approximately 1.0%.

During the initial 120 h, the 180 mL formulations generally retained a greater proportion of their initially absorbed water than the corresponding 250 mL formulations. The same pattern remained apparent after 720 h for poppy, rapeseed, and spruce. Birch represented an exception because birch 250 showed greater final retention than birch 180. Increasing the volume of citric acid solution therefore did not improve relative water retention consistently and, for most feedstocks, was associated with greater proportional water loss.

The differences between the two formulations cannot, however, be attributed solely to the volume or amount of citric acid. Increasing the solution volume from 180 to 250 mL simultaneously changed the liquid-to-solid ratio, absolute citric acid dose, mixing conditions, mass transfer, and subsequent drying behavior. The results should therefore be interpreted as differences between two operational formulations rather than as evidence of a direct effect of citric acid dose or crosslinking density.

The results also confirm that water absorption by a soil–biogel mixture after saturation and free drainage and relative water retention by an isolated biogel represent distinct properties. Poppy 180 produced the highest retained water mass in the soil–biogel experiment but did not show the highest relative retention during the initial 120 h. Conversely, spruce 180 showed comparatively high relative retention during this period despite its more moderate performance in the soil experiment. Initial water absorption and subsequent proportional water loss should therefore be evaluated separately.

The rapid initial decrease followed by slower water loss is consistent with desorption behavior previously reported for cellulose- and lignin-containing hydrogels [1,3,6,15]. Nevertheless, the present experiment did not characterize water-binding states, pore structure, matrix contraction, or internal morphology. The observed differences should consequently be interpreted as empirical feedstock–formulation responses rather than as direct evidence of differences in pore accessibility, network organization, or crosslinking density.

### 2.5. Integrated Interpretation of Feedstock Composition and Biogel Performance

No single measured feedstock constituent consistently explained both water absorption by the soil–biogel systems and relative water retention during climatic exposure. Poppy 180 produced the highest retained water mass after saturation and free drainage despite not having the highest cellulose content. Spruce 250 produced the second-highest value and was derived from the feedstock with the highest cellulose and lignin contents. Moreover, the two formulations prepared from the same feedstock sometimes differed substantially even though their initial chemical composition was identical. These observations indicate that feedstock composition alone was insufficient to predict biogel performance and that feedstock-specific properties interacted with the preparation conditions.

Cellulose contains hydroxyl groups that participate in hydrogen bonding with water and may participate in possible acid-mediated chemical modification [7,8,9]. Previous studies have demonstrated water-storage or moisture-regulation functions in hydrogels prepared from cellulose, cellulose nanofibers, cellulose cryogels, and rice-straw-derived cellulose [20,21,22,23]. Nevertheless, a greater cellulose content did not consistently correspond to greater water absorption in the present soil–biogel systems.

Spruce contained the highest proportion of cellulose, but the highest water absorption was recorded for poppy 180. Moreover, the two variants derived from the same feedstock sometimes differed considerably despite having the same initial cellulose content. Cellulose content alone therefore cannot explain the observed variation. The accessibility of hydroxyl groups, hemicellulose content, residual lignin, extractives, inorganic constituents, particle characteristics, and residual organic acids may all modify the contribution of cellulose to water uptake. However, the present data do not allow the individual effects of feedstock composition, acid incorporation, and physical structure to be separated.

Spruce, which contained the highest proportion of lignin, exhibited comparatively stable retention during the intermediate stage of climatic exposure. Unlike cellulose and hemicelluloses, lignin is relatively hydrophobic and is not expected to contribute strongly to initial water uptake. Its rigid aromatic structure may, however, influence the physical stability and drying behavior of lignocellulosic materials. Previous studies have shown that lignin-containing superabsorbent materials can be prepared from isolated biomass lignin, lignin–poly(vinyl alcohol) systems, and pulping black liquor [15,24,25,26,27]. These studies indicate that the contribution of lignin depends on its concentration, chemical functionality, method of incorporation, and interactions with the other polymer components.

Nevertheless, the role of lignin in the present whole-biomass materials cannot be isolated from the contributions of cellulose, hemicelluloses, extractives, and other feedstock constituents. Lignin content covaried with cellulose content and other feedstock characteristics preventing its independent contribution from being resolved. The comparatively high relative retention of spruce 180 during the initial 120 h is consistent with a possible contribution of its relatively lignified matrix. However, only four feedstocks were investigated, and the present results do not demonstrate that lignin directly controlled water retention.

**Figure 4:**
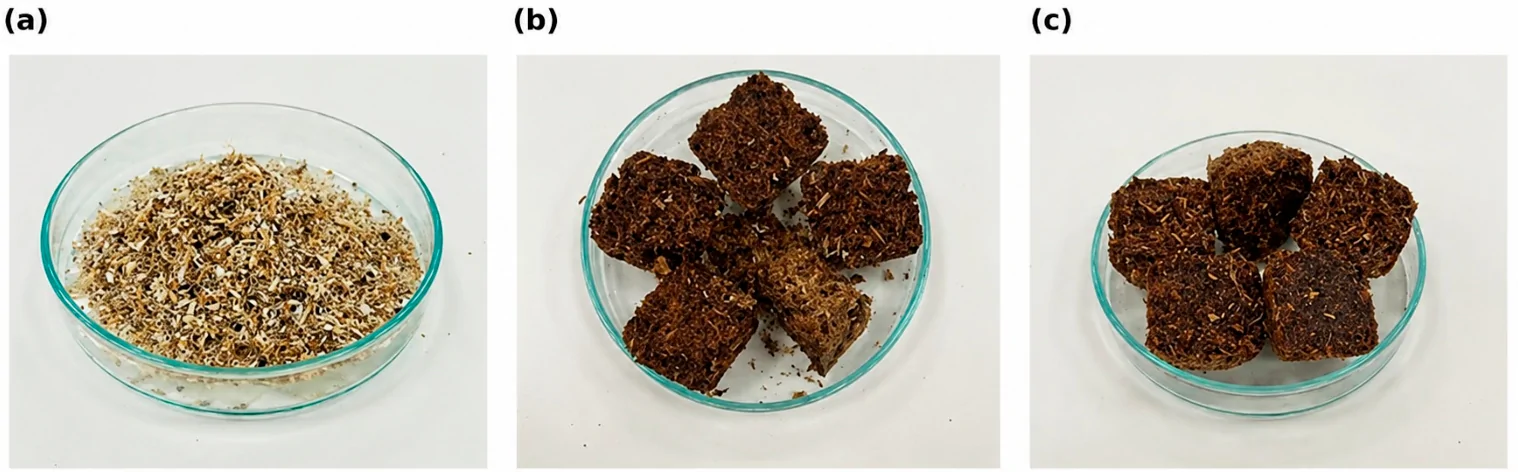
Representative macroscopic appearance of (**a**) untreated milled rapeseed straw and rapeseed-derived citric-acid-modified biogels prepared using (**b**) 180 mL and (**c**) 250 mL of 10 wt.% citric acid solution per 12 g of dry feedstock.

Feedstock density was excluded from the combined analysis. Wood density and the bulk density of agricultural residues were determined using different procedures and represent physically different quantities. Their numerical values are therefore not directly comparable and cannot be used as a common explanatory variable. Furthermore, neither measurement represents the porosity, pore-size distribution, or accessible surface area of the prepared biogels. Figure 4 and the associated regression equations were therefore removed from the revised manuscript.

The significant feedstock × formulation interaction observed for water absorption supports the conclusion that the response depended on the combination of feedstock and preparation conditions. Increasing the citric acid solution volume from 180 to 250 mL did not produce a uniform improvement across the four feedstocks. The higher-volume formulation increased the retained water mass in the spruce and rapeseed soil systems but reduced it in the corresponding birch and poppy systems. Relative retention during climatic exposure was generally higher for the 180 mL formulations, with birch representing an exception after 720 h. Because the two formulations differed simultaneously in solution volume, absolute citric acid amount, citric-acid-to-biomass ratio, and liquid-to-solid ratio, their contrasting behavior cannot be interpreted solely as an isolated effect of citric acid dose.

The comparison also highlights the difference between whole biomass biogels, and hydrogels prepared from purified polymers. Purified cellulose or lignin systems allow for more precise control over polymer concentration and chemical modification and network formation, whereas whole feedstocks contain interacting cellulose, hemicelluloses, lignin, extractives, and mineral components. Consequently, the performance of whole-biomass materials may be less predictable than that of purified-polymer hydrogels, but their preparation may require less feedstock fractionation and may facilitate the direct utilization of forestry and agricultural residues. Direct quantitative comparison remains difficult because previous studies used different polymers, preparation procedures, application rates, swelling media, and methods for calculating water absorption or retention [20,21,23,25,26,27].

Representative photographs of untreated milled rapeseed straw and the corresponding rapeseed 180 and rapeseed 250 formulations are presented in Figure 4. Compared with the untreated feedstock, the treated materials retained a heterogeneous particulate and fibrous appearance but were more cohesive and slightly darker, possibly as a result of the thermal treatment and subsequent drying.

The findings should be interpreted within the limitations of the experimental design. A single soil, distilled water, one biogel application rate, and one laboratory climatic regime were used. The effects of soil texture, pH, salinity, ionic strength, plant water uptake, biodegradation, and repeated wetting–drying cycles were not evaluated. In addition, the biogels were not washed after preparation, and their pH, residual acid content, gel fraction, equilibrium swelling ratio, morphology, and quantitative porosity were not determined. Residual citric and lactic acids may therefore have influenced both the FTIR spectra and water-sorption behavior.

Within these limitations, poppy 180 and spruce 250 were the most effective formulations for increasing the water retained by the tested soil after free drainage. Spruce 180 showed the highest relative retention during the initial 120 h, while poppy 180 and spruce 180 retained the largest residual proportions after 720 h. These results identify promising feedstock–formulation combinations for further evaluation but do not establish superiority over previously reported hydrogels or demonstrate performance under agricultural or field conditions. Further studies should include equivalent untreated controls, washed materials, pH and residual-acid measurements, isolated-biogel swelling and gel-fraction tests, morphological characterization, repeated wetting–drying cycles, and validation in different soil–plant systems.

## 3. Conclusions

This study showed that citric-acid-modified whole-biomass biogels prepared from Norway spruce, silver birch, poppy, and rapeseed residues differed markedly in water absorption and retention despite being produced under the same thermal conditions. Their performance was governed by the interaction between feedstock properties and formulation rather than by any single compositional parameter. The comparison therefore highlights the importance of evaluating chemically different whole-biomass feedstocks using the same preparation and testing procedures.

All biogel-containing systems retained more water than the untreated soil after saturation and free drainage at an application rate of approximately 3.1% dry biogel relative to dry soil mass. The highest value was obtained for poppy 180, which increased the retained water mass by 38.9% relative to the reference. Spruce 250 and poppy 250 also showed high values, corresponding to increases of 32.7 and 28.5%, respectively. The significant feedstock × formulation interaction demonstrated that increasing the volume of citric acid solution did not produce a uniform response among the tested feedstocks. Because solution volume, liquid-to-solid ratio, and absolute citric acid dose changed simultaneously, the observed response cannot be attributed to a single processing variable.

FTIR analysis showed changes in the carbonyl region consistent with the incorporation of carbonyl-containing acid components and possible ester formation within the lignocellulosic matrix. However, a stronger carbonyl contribution did not consistently correspond to higher water absorption or more stable retention. Because the whole-biomass biogels were compared with isolated cellulose, were not washed after preparation, and contained both citric and lactic acids, the FTIR results cannot be interpreted as direct evidence of covalent crosslinking or crosslinking density.

During cyclic climatic exposure, all biogels lost a substantial proportion of their initially retained water during the first 24 h, followed by slower desorption. Spruce 180 showed the highest relative retention during the initial 120 h, reaching approximately 39.0% after 120 h. After 720 h, poppy 180 and spruce 180 showed the highest residual retention, both approximately 5.5%. The 180 mL formulations generally retained a greater proportion of their initial water than the corresponding 250 mL formulations, although birch represented an exception after 720 h.

The results support further investigation of forestry and agricultural residues as renewable feedstocks for water-retaining soil amendments. Among the tested formulations, poppy 180 and spruce 250 provided the highest retained water mass in the soil–biogel experiment, while spruce 180 and poppy 180 showed the highest residual relative retention after climatic exposure.

These findings are limited to one soil, distilled water, one biogel application rate, and one laboratory climatic regime. Plant water availability, soil pH effects, salinity, ionic strength, biodegradation, and field performance were not evaluated. The results should therefore be considered laboratory screening rather than evidence of agricultural drought mitigation. Further work should include pH and residual-acid measurements, equivalent untreated and washed controls, isolated-biogel swelling and gel-fraction tests, repeated wetting–drying cycles, direct morphological characterization, and validation in soil–plant systems under controlled and field conditions.

## 4. Materials and Methods

### 4.1. Materials

Four lignocellulosic feedstocks representing two woody biomass sources and two agricultural post-harvest residues were used for biogel preparation:

• Norway spruce (*Picea abies* (L.) H. Karst.) sawdust;
• Silver birch (*Betula pendula* Roth) sawdust;
• Rapeseed (*Brassica napus* L.) straw;
• Poppy (*Papaver somniferum* L.) straw.

All four feedstocks were obtained from long-term research material stocks maintained at the Faculty of Forestry and Wood Sciences, Czech University of Life Sciences Prague, Czech Republic. The woody feedstocks consisted of sawdust generated during previous laboratory wood processing, whereas rapeseed and poppy straw represented archived post-harvest residues. No surface coating, chemical impregnation, or additional chemical pretreatment was applied before biogel preparation. All feedstocks were mechanically processed and dried before biogel preparation.

Citric acid (anhydrous, Penta Chemicals, Prague, Czech Republic) was dissolved in distilled water to prepare a 10 wt.% aqueous solution. Lactic acid (80% p.a.; Penta Chemicals, Prague, Czech Republic) was used as a processing additive and was added at the same fixed amount to all formulations. Distilled water was used for biogel preparation and all water-absorption and retention experiments.

The soil used in the soil–biogel water-absorption experiment was a Cambisol obtained from an experimental site near Kostelec nad Černými Lesy, Czech Republic. Before use, the soil was air-dried and homogenized by passing it through a 0.5 mm sieve. Its approximate pH was 6.5, and its electrical conductivity ranged from 350–500 µS cm^−1^. A detailed particle-size distribution was not available. No fertilizer or other amendment was added before the experiment.

### 4.2. Mechanical Treatment of the Feedstocks

The lignocellulosic feedstocks were ground separately using an MF 10 BASIC knife mill (IKA, Staufen, Germany). Spruce and birch sawdust were milled for 2 min, whereas rapeseed and poppy straw were milled for 1 min to limit the excessive production of fine particles.

After grinding, the materials were sieved through a laboratory sieve, and the fraction with a particle size of <5 mm was collected for biogel preparation. The selected fractions were subsequently dried at 45 °C for 24 h and stored in closed containers under dry laboratory conditions until further use. The same particle-size fraction and drying procedure were applied to both formulations of each feedstock.

### 4.3. Selected Chemical Composition of the Feedstocks

The selected chemical constituents of each feedstock were determined in triplicate and expressed on an oven-dry biomass basis. Cellulose content was determined using the Seifert method [28], while acid-insoluble lignin was measured according to the Klason method [29]. Holocellulose was determined according to Wise et al. [30], and hemicellulose content was calculated as the difference between holocellulose and cellulose contents. Extractives and ash were determined according to the relevant TAPPI methods [31,32], with extractives representing the acetone-soluble.

The individual constituents were determined using separate analytical procedures and were not treated as a closed summative analysis. Consequently, their total was not expected to equal exactly 100% because of method-specific recovery, analytical overlap, and biomass components not quantified by the selected procedures.

### 4.4. Preparation of Citric-Acid-Modified Lignocellulosic Biogels

Two formulations differing in the volume of 10 wt.% citric acid solution were prepared for each feedstock:

• 12 g of dry feedstock and 180 mL of citric acid solution;
• 12 g of dry feedstock and 250 mL of citric acid solution.

These quantities corresponded to solution-to-feedstock ratios of 15.0 and 20.8 mL g^−1^, respectively. Because the citric acid concentration was kept constant, increasing the solution volume simultaneously increased the liquid-to-solid ratio and the absolute amount of citric acid. The comparison therefore represented two operational formulations and was not designed to isolate the effect of a single chemical variable.

The feedstock and citric acid solution were heated at 100 °C for 30 min under continuous stirring at 250 rpm. Five milliliters of 80% lactic acid were added 10 min before the end of the heating period. Lactic acid was used as a fixed processing co-modifier in all formulations to improve homogenization of the biomass suspension]. Its amount was not varied, and its individual contribution was not evaluated using a citric-acid-only control.

After heating, the mixtures were stirred at laboratory temperature 90–95 °C for a further 3 h. During this stage, the mixtures formed heterogeneous suspensions. The resulting materials were transferred into molds and dried at 90 °C for 24 h. The dried biogels were stored in a desiccator until constant mass.

The materials were not washed after acid treatment. Consequently, unreacted citric and lactic acids may have remained in the dried products. This limitation was considered when interpreting the FTIR spectra and water-sorption results.

Samples were designated by feedstock and solution volume, for example, poppy 180 and spruce 250. For each formulation, one production batch subsequently divided into five measurement samples was prepared.

Macroscopically, the dried products retained the heterogeneous particulate or fibrous character and general physical consistency of the corresponding raw feedstocks. Following acid treatment, the particles were bound into cohesive agglomerates. The products were opaque and slightly darker than the untreated feedstocks, which was likely associated with thermal exposure during heating and drying. No substantial visual differences in consistency were observed between the corresponding 180 and 250 mL formulations.

### 4.5. FTIR Spectroscopy and Carbonyl-Region Index

FTIR spectroscopy was used to compare cellulose isolated from each feedstock with the corresponding acid-modified whole-biomass biogels prepared using 180 and 250 mL of 10 wt.% citric acid solution. The isolated cellulose samples were used as compositional references rather than as chemically equivalent untreated controls because the biogels also contained hemicelluloses, lignin, extractives, inorganic constituents, and residual processing acids.

The spectra were recorded using a Nicolet iS20 FTIR spectrometer (Thermo Fisher Scientific, Waltham, MA, USA) equipped with an attenuated total reflectance accessory containing a diamond crystal. Each spectrum was obtained in absorbance mode by accumulating 64 interferograms at a spectral resolution of 4 cm^−1^ over the wavenumber range of 4000–400 cm^−1^. A background spectrum was collected before each measurement. The samples were measured directly at laboratory temperature, and one spectrum was obtained for each cellulose and biogel sample. Consequently, the FTIR results provide no estimate of analytical variability.

Before comparison, the spectra were baseline-corrected and normalized to the polysaccharide C–O stretching region. The main spectral regions evaluated included O–H stretching at 3600–3000 cm^−1^, C–H stretching at 2950–2850 cm^−1^, carbonyl stretching in 1760–1680 cm^−1^, and C–O and C–O–C stretching at 1200–1000 cm^−1^.

Because the biogels were not washed after preparation and lactic acid was added to all formulations, absorbance in the carbonyl region could include contributions from native whole-biomass components, residual citric and lactic acids, free carboxylic groups, and possible ester bonds. The FTIR spectra were therefore not used as direct evidence of covalent crosslinking.

To compare the relative absorbance contribution in the carbonyl region semiquantitatively, a carbonyl-region index was calculated as follows:

(1) $$CI\;=\;\frac{A_{1760-1680}}{A_{1080-1000}},$$

where *A*_1760−1680_ is the baseline-corrected integrated absorbance area in the carbonyl region and *A*_1080−1000_ is the integrated absorbance area of the polysaccharide C–O stretching region. The carbonyl-region index was interpreted as a semiquantitative indicator of the relative contribution of carbonyl-containing structures and not as a direct measure of esterification or covalent crosslinking density. One FTIR spectrum was evaluated for each sample, and the index was therefore reported descriptively without standard deviation or inferential statistical analysis.

### 4.6. Water Absorption After Free Drainage of Soil–Biogel Systems

The amount of water retained by the soil–biogel systems after saturation and free drainage was determined gravimetrically. Approximately 5 g of dry biogel was thoroughly mixed with approximately 160 g of air-dried and homogenized soil. The dry biogel-to-soil mass ratio ranged from 3.12 to 3.19%, corresponding to an application rate of approximately 3.1% (*w*/*w*). Untreated soil without biogel addition was used as the reference. The exact masses of soil and biogel were recorded for each replicate.

For each treatment, five soil–biogel measurement units were assembled separately using individual portions of the corresponding biogel and soil. These units represented replicate measurements of the soil–biogel system rather than five independently synthesized biogel batches.

The prepared systems were saturated with distilled water under free-drainage conditions. Distilled water was added gradually until complete saturation and visible free drainage were achieved. After saturation, excess water was allowed to drain, and the samples were weighed using a laboratory balance with a precision of 0.01 g.

The amount of water retained after free drainage was determined as the difference between the saturated mass and the initial mass of the dry soil–biogel system:

(2) $$W_{ret}\;=\;m_{sat}-\left(m_{soil}\;+\;m_{biogel}\right),$$

where *W*_ret_ is the mass of water retained after free drainage, *m*_sat_ is the saturated mass of the soil–biogel system, *m*_soil_ is the initial dry soil mass, and *m*_biogel_ is the initial dry biogel mass. For the untreated reference, *m*_biogel_ = 0.

The percentage increase in retained water mass relative to the untreated soil was calculated from the mean retained-water values:

(3) $$Increase\;=\;\frac{W_{treatment}\;-\;W_{reference}}{W_{reference}}\cdot 100,$$

All measurements were performed using five independent replicates separately assembled replicate soil systems (*n* = 5), and the results were expressed as mean ± standard deviation. This measurement describes water retention by the complete soil–biogel system after free drainage and does not represent the equilibrium swelling ratio of the isolated biogel.

### 4.7. Relative Water Retention Under Simulated Climatic Conditions

The relative water retention of the prepared lignocellulosic biogels was evaluated under cyclic temperature and relative-humidity conditions using a Weiss climatic chamber (Weiss Technik, Prague, Czech Republic). Before climatic exposure, the dry biogel samples were saturated with distilled water under free-saturation conditions and weighed to determine their initial saturated mass. The dry mass of each sample was recorded before saturation.

A repeated 24 h climatic cycle was applied as a laboratory-defined regime covering temperature and relative-humidity conditions occurring during summer in Central Europe. The regime was not intended to reproduce one specific meteorological day and consisted of the temperature and relative-humidity conditions listed in Table 5.

The individual temperature and relative-humidity values represented programmed chamber setpoints. Transitions between consecutive setpoints were controlled automatically by the climatic chamber rather than being changed instantaneously. Air circulation was provided by the internal forced-air system of the chamber; the exact airflow velocity was not recorded.

The samples were placed on a sand layer inside the climatic chamber with sufficient spacing to permit air circulation. Their positions were not randomized or systematically changed during the experiment. At each weighing time, the samples were removed from the chamber, weighed, and returned to approximately the same positions on the sand layer.

The samples were removed from the climatic chamber and weighed after 0, 24, 96, 120, and 720 h. After each weighing, they were immediately returned to the chamber. The total exposure period was 720 h, corresponding to 30 repeated 24 h climatic cycles. No intermediate weighing was performed between 120 and 720 h.

Relative water retention at each weighing time was calculated as follows:

(4) $${WR}_{t}=\frac{m_{t}{\;-\;m}_{d}}{m_{0}\;-\;m_{d}}\cdot 100,$$

where *WR*_t_ is the relative water retention at time *t*, *m*_t_ is the sample mass at time *t*_1_, *m*_d_ is the initial dry biogel mass, and *m*_0_ is the initial saturated sample mass. According to this definition, all formulations had a relative retention value of 100% at 0 h.

All measurements were performed using five replicate sample (*n* = 5), and the retention curves were presented as normalized mean values. The climatic-chamber experiment quantified proportional water loss from separately saturated biogels and was evaluated independently from the soil–biogel free-drainage experiment.

### 4.8. Statistical Analysis

All quantitative results are presented as mean ± standard deviation, unless stated otherwise. Chemical composition analyses were performed in triplicate, whereas water absorption by the soil–biogel systems, and relative water-retention measurements were conducted using five replicate measurement units (*n* = 5) for each treatment. For the water experiments, *n* = 5 refers to separately measured samples prepared from the corresponding biogel formulation and not to five independently synthesized production batches.

Relative water retention was calculated separately for each replicate sample before calculation of the treatment mean. The FTIR carbonyl-region index was based on one spectrum per sample and was therefore reported descriptively without standard deviation or inferential statistical analysis.

The effects of feedstock type and formulation on water retained by the soil–biogel systems after free drainage were evaluated using two-way analysis of variance. Feedstock and formulation (180 or 250 mL) were treated as fixed factors. The untreated reference soil was excluded from the factorial analysis because it did not correspond to either level of the formulation factor.

Differences among the biogel-containing treatments were evaluated using Tukey’s HSD post hoc test. Statistical significance was accepted at *p* < 0.05. Partial eta squared (ηp^2^) was reported as a measure of effect size for the main effects and their interaction. The analysis was performed using the replicate soil–biogel measurements rather than treatment mean.

Regression analyses relating feedstock composition or density to biogel performance were removed. Only four independent feedstock compositions were available, and replicate performance measurements did not constitute independent observations of cellulose or lignin content. In addition, wood density and agricultural-residue bulk density represented different physical quantities and were not directly comparable. Relationships between feedstock composition and performance were therefore discussed descriptively.

The relative water-retention profiles were also evaluated descriptively because the purpose was to compare water-loss patterns among formulations at the selected weighing times. No inferential statistical analysis was applied to the reconstructed retention curves.

Statistical analyses were performed using Statistica, version 14.1 (TIBCO Software Inc., Palo Alto, CA, USA), while graphical processing was conducted using Microsoft Excel (Microsoft Corporation, Redmond, WA, USA). The revised relative-retention figure was prepared from normalized treatment means.

## Figures and Tables

**Table 1:** Selected chemical constituents of the lignocellulosic feedstocks.

Feedstock	Ash, %	Extractives, %	Cellulose, %	Lignin, %	Hemicelluloses, %
Birch	0.69 ± 0.03	2.71 ± 0.07	38.70 ± 0.62	19.21 ± 0.33	24.13 ± 0.49
Poppy	1.67 ± 0.05	7.53 ± 0.15	35.16 ± 0.48	22.70 ± 0.37	34.54 ± 0.58
Rapeseed	4.45 ± 0.08	5.31 ± 0.12	33.90 ± 0.54	21.35 ± 0.42	42.25 ± 0.63
Spruce	0.42 ± 0.02	1.33 ± 0.04	47.90 ± 0.71	28.90 ± 0.51	21.40 ± 0.66

Values are expressed as mean ± standard deviation based on three analytical replicates (*n* = 3). The reported parameters represent independently determined constituents rather than a closed summative analysis. Hemicellulose content was calculated as the difference between holocellulose and cellulose, whereas extractives represent the acetone-soluble fraction determined using the specified TAPPI method. Consequently, the sum of the reported constituents is not necessarily equal to 100% because of method-specific recovery, analytical overlap, and unquantified biomass components.

**Table 2:** Main FTIR bands considered in the interpretation of cellulose and lignocellulosic biogels.

Spectral Region (cm^−1^)	Main Assignment	Relevance to the Present Study
3600–3000	O–H stretching	Hydrogen bonding and availability of hydroxyl groups
2950–2850	C–H stretching	Aliphatic groups of polysaccharides and residual lignocellulosic components
1760–1680	C=O stretching	Overlapping contributions from ester carbonyls, free carboxylic groups, native biomass components, and residual citric and lactic acid
1650–1600	Bound water and/or aromatic vibrations	Water associated with the matrix and contribution of residual lignin
1450–1300	C–H deformation and O–H bending	Structural changes in polysaccharides
1200–1000	C–O and C–O–C stretching	Polysaccharide backbone; region used for spectral normalization
Approximately 895	β-glycosidic linkages	Characteristic cellulose structure

Band assignments are based on the spectra obtained in this study and published data for cellulose and organic-acid-modified polysaccharide lignocellulosic materials. Exact peak positions may vary among feedstocks and formulations, and individual bands may contain overlapping contributions from several chemical components.

**Table 3:** Descriptive carbonyl-region index obtained for isolated cellulose and acid-modified whole-biomass biogels.

Feedstock	Cellulose	Biogel 180	Biogel 250
Birch	0.836	1.890	2.099
Spruce	0.051	1.631	0.982
Poppy	0.385	0.347	0.337
Rapeseed	0.502	0.710	0.737

The index was calculated as the ratio of integrated absorbance areas at 1760–1680 and 1080–1000 cm^−1^ after baseline correction. Values are based on one FTIR spectrum per sample and are therefore presented without standard deviations. The values provide no estimate of analytical variability. Comparisons between isolated cellulose and whole-biomass biogels should be interpreted cautiously because the materials are not compositionally equivalent.

**Table 4:** Water absorption after saturation and free drainage of soil–biogel systems containing approximately 3.1% dry biogel relative to dry soil mass.

Treatment	Biogel Mass (g)	Soil Mass (g)	Water Retained After Free Drainage (g)	Increase in Retained Water Relative to Reference Soil (%)
Reference soil	0	160.05 ± 0.21	88.46 ± 0.38 ^g^	0.0
Birch 180	5.08 ± 0.08	160.15 ± 0.13	106.72 ± 1.27 ^d^	20.6
Birch 250	5.07 ± 0.06	160.22 ± 0.19	97.30 ± 1.11 ^f^	10.0
Poppy 180	5.05 ± 0.02	160.01 ± 0.08	122.86 ± 2.07 ^a^	38.9
Poppy 250	5.11 ± 0.09	160.04 ± 0.16	113.66 ± 1.53 ^c^	28.5
Rapeseed 180	5.03 ± 0.03	160.70 ± 0.57	100.13 ± 1.22 ^ef^	13.2
Rapeseed 250	5.04 ± 0.11	160.74 ± 0.42	106.13 ± 0.98 ^d^	20.0
Spruce 180	5.03 ± 0.07	160.57 ± 0.39	102.01 ± 1.34 ^e^	15.3
Spruce 250	5.02 ± 0.04	160.32 ± 0.18	117.35 ± 1.69 ^b^	32.7

Values are expressed as mean ± standard deviation based on five replicate soil–biogel systems (*n* = 5). The biogel dose was calculated on a dry-mass basis and ranged from 3.13 to 3.19% relative to the dry soil mass. Different superscript letters in the retained-water column indicate significant differences among the eight biogel-containing treatments according to Tukey-adjusted post hoc comparisons at *p* < 0.05. The percentage increase relative to the reference soil was calculated from the mean retained-water values. The untreated reference was not included in the factorial analysis or post hoc comparison.

**Table 5:** Temperature and relative-humidity conditions applied during the repeated 24 h climatic cycle.

Time	Temperature of Air, °C	Air Relative Humidity, %
0–2 h	17	60
2–4 h	15	75
4–6 h	12	60
6–8 h	19	45
8–12 h	27	30
12–17 h	35	25
17–21 h	25	35
21–24 h	21	55

## Data Availability

The data presented in this study are available on request from the corresponding author due to (internal project).
